# The relation between hemispheric lateralisation and measures of immune competence and adherence in Human Immunodeficiency Virus Type 1 (HIV-1)

**DOI:** 10.1186/1742-4690-9-S1-P79

**Published:** 2012-05-25

**Authors:** Rachel C Sumner, Alexander V Nowicky, Andrew Parton, Carolien Wylock, Renata Cserjesi, Patrick Lacor, Yori Gidron

**Affiliations:** 1 Brunel University, Manchester, UK

## Introduction

Communication from the brain to the immune system is influenced by hemispheric lateralisation (HL). Left-HL is immunopotentiating, right-HL is immunosuppressive. Only one study has examined the effects of HL on the progression of HIV (Gruzelier et al., 1996). That study included a small sample with very little control over third variables. The present study tested whether left HL predicted higher CD4 and CD8 levels, statistically controlling for confounders.

## Methods

Employing two neuropsychological assessments of HL (line bisection task and Zenhausern’s Hemispheric Preference Test), 69 HIV-1+ patients were followed prospectively. Numerous exclusion criteria and confounder assessments were employed (e.g., age, sex, mode of contraction, medication adherence) to provide a more rigourous and controlled analysis.

## Results

The present work corroborated the theory of asymmetrical influence on HIV immunity by HL via a moderator: ethnicity. The main analysis of the research findings did not attain statistical significance in the whole group of patients. However, among African patients, left-HL predicted better immunity, while no such relationship was seen in European patients, independent of confounders. Further observations were made between HL and HIV-relevant behaviours. Left HL was related to higher number of sexual partners in Europeans. A near-significant relationship was observed between left HL and longer periods between HIV clinic attendances in Africans.

## Conclusions

The present study adds new information concerning a moderating factor of the HL-immunity relationship in HIV. As expected, left-HL predicted higher CD4 and CD8 counts, but only in African patients. Further, the added methodological and statistical control employed, extend the validity of the HL-immunity relationship. Moreover, the present study has uncovered behavioural implications of HL in HIV disease. Potential explanations for neurobiological pathways in the relationship between HL and immunity are discussed.

